# Microinjection of antisense oligonucleotides into living mouse testis enables lncRNA function study

**DOI:** 10.1186/s13578-021-00717-y

**Published:** 2021-12-17

**Authors:** Zhaohui Chen, Li Ling, Xiaolian Shi, Wu Li, Huicong Zhai, Zhenlong Kang, Bangjin Zheng, Jiaqi Zhu, Suni Ye, Hao Wang, Lingxiu Tong, Juan Ni, Chaoyang Huang, Yang Li, Ke Zheng

**Affiliations:** 1 State Key Laboratory of Reproductive Medicine, Nanjing Medical University, 211166 Nanjing, China; 2 Department of Obstetrics and Gynecology, the Affiliated Hospital of Hangzhou Normal University, 310015 Zhejiang, China; 3 Department of Cardiology, the First Affiliated Hospital, Zhejiang University School of Medicine, 310014 Zhejiang, China

**Keywords:** lncRNA (long non-coding RNA), *Tsx*, ASO (antisense oligonucleotide), Knock down, Testis injection

## Abstract

**Background:**

Long non-coding RNAs (lncRNAs) have been the focus of ongoing research in a diversity of cellular processes. LncRNAs are abundant in mammalian testis, but their biological function remains poorly known.

**Results:**

Here, we established an antisense oligonucleotides (ASOs)-based targeting approach that can efficiently knock down lncRNA in living mouse testis. We cloned the full-length transcript of lncRNA *Tsx* (testis-specific X-linked) and defined its testicular localization pattern. Microinjection of ASOs through seminiferous tubules in vivo significantly lowered the *Tsx* levels in both nucleus and cytoplasm. This effect lasted no less than 10 days, conducive to the generation and maintenance of phenotype. Importantly, ASOs performed better in depleting the nuclear *Tsx* and sustained longer effect than small interfering RNAs (siRNAs). In addition to the observation of an elevated number of apoptotic germ cells upon ASOs injection, which recapitulates the documented description of *Tsx* knockout, we also found a specific loss of meiotic spermatocytes despite overall no impact on meiosis and male fertility.

**Conclusions:**

Our study detailed the characterization of *Tsx* and illustrates ASOs as an advantageous tool to functionally interrogate lncRNAs in spermatogenesis.

**Supplementary Information:**

The online version contains supplementary material available at 10.1186/s13578-021-00717-y.

## Background

Of general curiosity, nearly 98% of the human genome does not have coding functions [1, 2]. Long non-coding RNAs (lncRNAs) are non-protein coding RNAs longer-than-200 nt which may evolve a regulatory function from ‘junk’ transcripts [3–5]. With the rapid identification of lncRNAs through deep sequencing comes the urgent demand for the characterization of their biological functions [6–8]. In mammals, transcription of lncRNAs is shown more widespread and dynamic in testis than other organs [9, 10]. Numerous novel lncRNA genes were discovered in spermatogenic cells, rapidly outpacing the rate of characterizing them [11–14]. Reasons behind this gap include the infrequent emergence of loss-of-function phenotype of lncRNAs as well as the lack of an ideal culturable germline affordable to large-scale lncRNA functional screening [15–17].

Employment of various methods such as CRISPR/Cas9-mediated genome editing, RNA interference (RNAi) or antisense oligonucleotides (ASOs) have been reported in lncRNA loss-of-function studies [18–21]. Although the CRISPR/Cas9 system removes transcripts thoroughly compared with the knockdown, it’s more laborious, costly and time-consuming. Also, CRISPR/Cas9 is not applicable in some cases. Unlike mRNA, lncRNA function is not prone to be influenced by small deletions or insertions [18, 22]. Moreover, genetic manipulation of lncRNAs is likely to perturb the overlapping or adjacent genes. To circumvent such problems, in vivo local knockdown by means of short-hairpin RNAs (shRNAs), small interfering RNAs (siRNAs) or ASOs may be a good alternative. In recent reports, testis transduction of shRNAs in adeno-associated viral (AAV) or lentiviral vectors are demonstrated successful in knockdown of either mRNAs [23, 24] or lncRNAs [14, 25]. siRNA microinjection to achieve transient knockdown effect in young testis has proved a simplified method to examine the meiotic function of protein-coding genes [26]. piRNAs can be targeted by ASOs with the aid of electric transduction in testis [23]. Meanwhile, both siRNAs [27, 28] and ASOs [29–31] have been used for lncRNA knockdown in vivo other than in mouse testis.

ASOs, differing from siRNAs, are synthesized oligonucleotides with a length of 15–20 nt that can hybridize complementarily with an endogenous RNA to form DNA/RNA heteroduplex and then induce recruitment of the nucleus-enriched RNase H followed by target RNA cleavage [18, 32]. Pharmacokinetic studies in mice showed that ASOs have long half-lives that can last several weeks [33–35]. Interestingly, nucleus-localized lncRNAs are more easily targeted by ASOs. In knockdown assays on lncRNAs with different subcellular localization, the knockdown efficiency of ASOs for lncRNAs that primarily located in nucleus is higher than that of siRNAs [36]. Moreover, siRNAs-mediated knockdown affects lncRNAs expression solely at post-transcriptional level, while ASOs can also target upon nascent transcripts and block transcription [37]. In some cases, the transcription or splicing process from the transcribed locus rather than the mature lncRNA products confers the lncRNA functionality [7, 38, 39]. Degradation of transcripts carried out by ASOs, rather than siRNAs, can clarify the lncRNA function in its nascent or on-transcription form [37, 40]. As attractive as the ASO tool is for envisioning its being equivalently applicable in lncRNA knockdown in mammalian testis, a methodological establishment awaits a pioneering attempt.

Considering this technically practical idea a driver of many future investigations to understand lncRNA biological roles in spermatogenesis, we aimed to set up a framework initiated from validating the knockdown effectiveness of testis injection by ASOs through completing a battery of standard phenotypic examinations. To explore ASO application conditions, we first selected a widely expressed, highly nucleus-enriched lncRNA *Malat1* [41]. For extending to function study, we knocked down a testis-specific lncRNA *Tsx*, which represents one rarely documented lncRNA yielding observable phenotype when knocked out in vivo [42, 43]. We defined localization of *Tsx* and characterized its potential function in sustaining the pool of meiotic germ cells. Comparative assessment of ASOs and siRNAs targeting *Tsx* highly recommends the former as a better tool for lncRNA function study in testis.

## Results

### ASOs-mediated lncRNA knockdown is dose-dependent

**Fig. 1:**
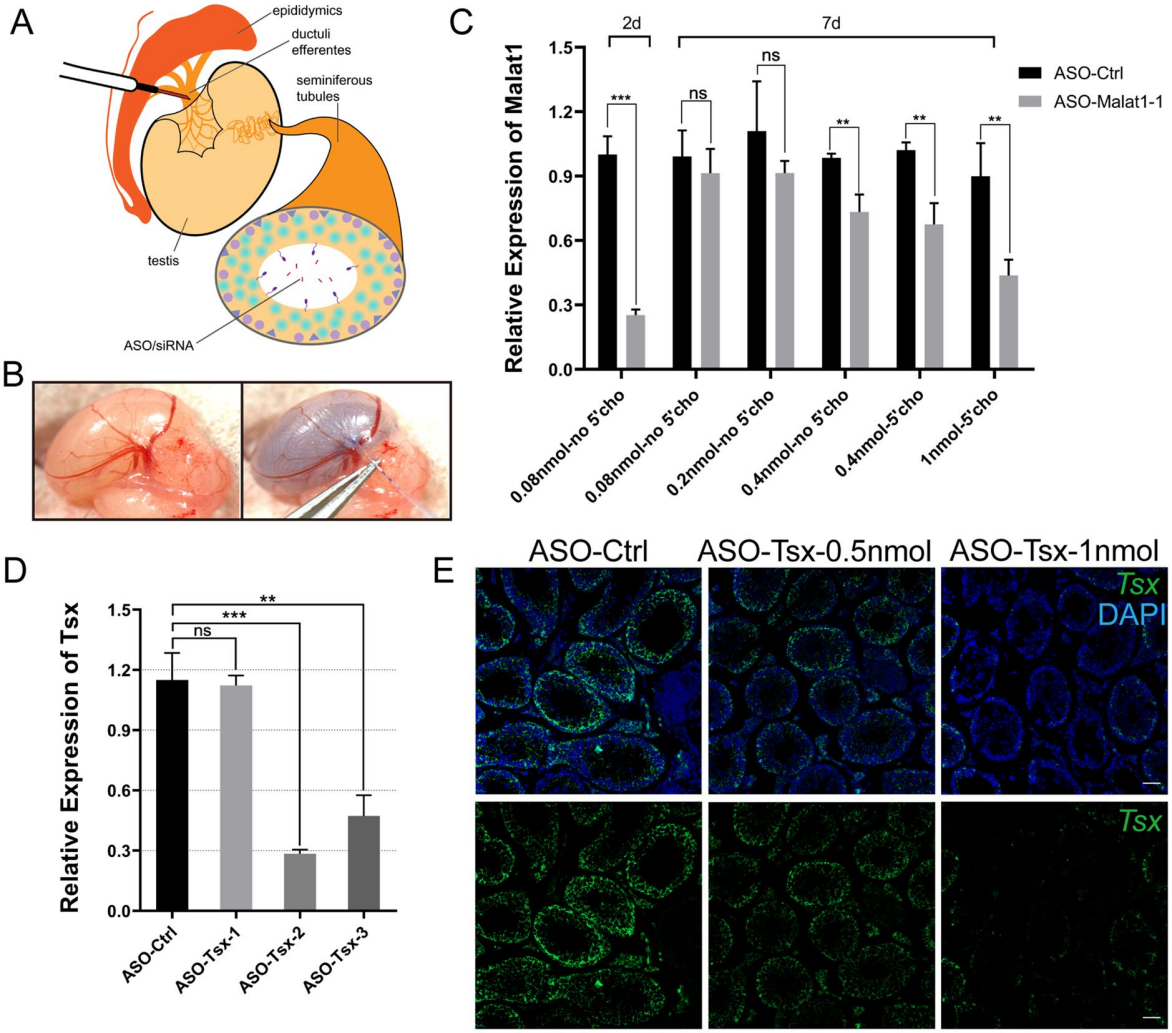
Knockdown efficiency of ASOs in testis. **A** Schematic diagram of microinjection process through seminiferous tubules. **B** Photos of testis from 3-week-old mouse taken before (left) and after (right) injection. Filling of seminiferous tubules was monitored with the help of Trypan blue. **C** Relative expression of *Malat1* detected by RT-PCR after injecting different doses of ASO-*Malat1*-1 into mouse testis. Testes were collected 2 or 7 days after injection. Values are expressed as mean ± SD. Statistical significance was determined using *t* tests. **p < 0.01 and ***p < 0.001. n = 3. **D** Relative expression of *Tsx* in testis measured by RT-PCR 2 days after injection of three different ASOs. Values are expressed as mean ± SD. Statistical significance was determined using *t* tests. **p < 0.01 and ***p < 0.001. n = 3. **E** *Tsx* RNA (green) detected by FISH in testes injected by different doses of ASO-*Tsx-2*. Scale bar, 50 μm

To obtain the optimal ASO injection conditions, we started to inject testis with *Malat1* (Metastasis associated lung adenocarcinoma transcript 1) which is highly expressed in GC-2 cells and mouse testis [41]. We first examined the knockdown efficiency of two designed ASOs targeting *Malat1* transcripts in GC-2 cells by performing fluorescence in situ hybridization (FISH) two days after infection. FISH results showed that the number of *Malat1*-positive cells was significantly reduced in GC-2 cells transfected with ASO-*Malat1*-1 (Additional file 1: Fig. S1). Subsequently, we injected ASO-*Malat1*-1 into testes of 3-week-old mice and expression of *Malat1* was measured by RT-PCR at post-injection 2 and 7 days, respectively. The dilutions of ASOs were directly injected from the ductuli efferentes into seminiferous tubules (Fig. 1A). ASOs with Trypan blue as an indicator filled nearly two third of seminiferous tubules after injection (Fig. 1B). Results of RT-PCR showed that nearly 80% knockdown efficiency was tested after 2 days of injection at the dosage of 0.08 nmol (Fig. 1C). However, after 7 days, the expression of *Malat1* returned to normal levels (Fig. 1C). To maintain the knockdown effect for a longer period of time, we increased the ASO dosage of initial injection. A maximum volume (4 µL) of higher amounts of ASOs (0.2-1 nmol) were injected into the testis. More than 1 nmol ASOs would become too viscous to be injected fluently. We collected mouse testis 7 days later for RT-PCR test and determined the range of 0.4-1 nmol ASOs that maintained the knockdown efficacy, with no use for cholesterol modification on ASOs (Fig. 1C).

Next, we turned to test another lncRNA *Tsx* (testis-specific X-linked). We started with cloning the full-length *Tsx* by 5’-end and 3’-end rapid amplification of cDNA ends (RACE) and identified a 818-nt transcript in mouse testis (Additional file 1: Fig. S2). Three different *Tsx*-targeting ASOs were injected, among which ASO-*Tsx*-2 was adopted for its highest knockdown efficiency (Fig. 1D). To confirm the best amount for ASO injection, we compared two different amounts (0.5 nmol and 1 nmol) and assessed separately their 10-day knockdown efficiency by FISH. Nearly half tubules of testes injected at 0.5 nmol showed no reduction of *Tsx*, while over 80% of the tubules showed dramatic reduction at 1 nmol (Fig. 1E). No obvious artificial effect was observed between control and *Tsx*-knockdown testis sections. Therefore, we selected 1 nmol ASO-*Tsx*-2 for subsequent experiments.

### *Tsx* is distributed in both nucleus and cytoplasm

**Fig. 2:**
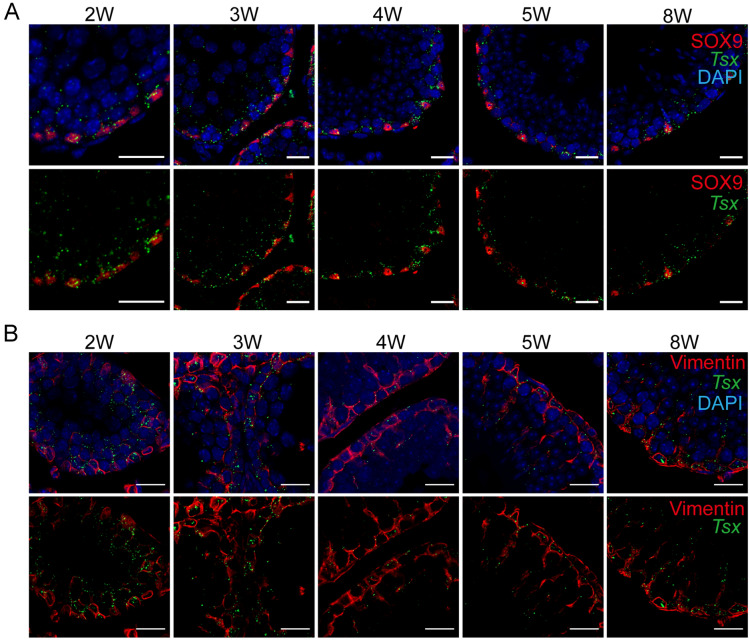
Localization pattern of *Tsx* in testis. **A** *Tsx* RNA (green) co-staining with nuclei marker of Sertoli cells SOX9 (red). Testes collected from 2 to 8 week-old (W) mice were fixed in 4% PFA, paraffin embedded and then sectioned. Nuclei were stained with DAPI. Scale bar, 20 μm. **B** *Tsx* RNA (green) co-staining with the cytoplasm marker of Sertoli cells Vimentin (red). Nuclei were stained with DAPI. Testes were treated the same as that in **A**. Scale bar, 20 μm

Before further evaluating knockdown effect on *Tsx*, we characterized its expression and localization in postnatal testes. RT-PCR showed that *Tsx* began to express stably at high levels from 2nd week (2 W) after birth through the 8-week-old (8 W) adulthood. (Additional file 1: Fig. S3A). We then performed co-localization assays by *Tsx* FISH in combination with SOX9 immunofluorescence (IF) on seminiferous tubule sections. The results showed that the punctate signals of lncRNA *Tsx* were partially within the SOX9-positive nucleus of Sertoli cells (Fig. 2A). The rest of *Tsx* signals spread over from the basal lamina toward lumen, possibly along the cytoplasmic area of Sertoli cells which spaces between germ cells. This speculation was confirmed by co-staining of the Sertoli cytoplasmic marker Vimentin (Fig. 2B). Clearly, *Tsx* were also shown as individual dots within the SYCP3-marked nucleus of spermatocytes in 2-week-old testis (Additional file 1: Fig. S3B, indicated by arrows). Moreover, only a few *Tsx* signals overlapped with the germ cell marker DDX4 (Additional file 1: Fig. S3C, indicated by arrows), consolidating that the majority of *Tsx* molecules located in the cytoplasmic territory of Sertoli cells but not that of germ cells. Together, *Tsx* is a nucleocytoplasmic lncRNA in testis.

### ASOs, but not siRNAs, persistently inhibit *Tsx* expression in vivo

**Fig. 3:**
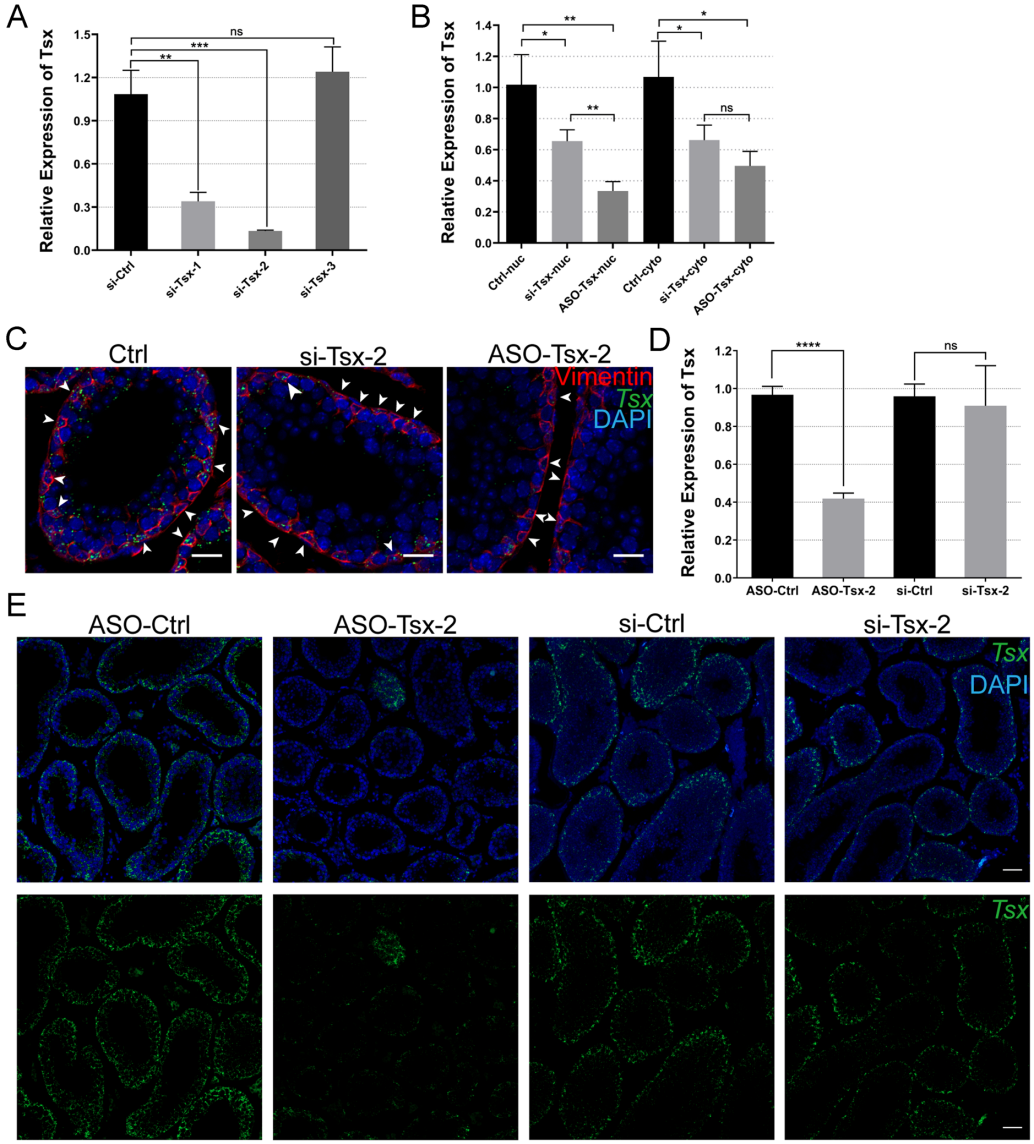
Comparative evaluation of ASOs and siRNAs in testis. **A** The RNA level of *Tsx* detected by RT-PCR in control and knockdown testis treated with three different siRNAs. **p < 0.01, ***p < 0.001, n = 3. **B** RT-PCR analysis of *Tsx* expression in cytoplasm and nucleus 2 days after injection of si-*Tsx*-2 or ASO-*Tsx*-2. *p < 0.05, **p < 0.01, n = 3. **C** FISH of *Tsx* RNA (green) with IF of Vimentin (red). Testes were collected 2 days after injection of siRNAs or ASOs. Nuclei were stained with DAPI. Nuclei of Sertoli cells were indicated by white arrows. Scale bar, 20 μm. **D** RT-PCR results of *Tsx* RNA level 10 days after injection of si-*Tsx*-2 or ASO-*Tsx*-2. ****p < 0.0001, n = 3. **E** FISH images of *Tsx* RNA in testes 10 days after injection of si-*Tsx*-2 or ASO-*Tsx*-2. Nuclei were stained with DAPI. Scale bar, 50 μm. **A**, **B**, **D** Values are expressed as mean ± SD. Statistical significance was determined using *t* tests

Next, we designed three pieces of siRNAs sequences, and selected one with highest knockdown efficiency for a comparison with ASOs (Fig. 3A). Two days after injection into 3-week-old testes, preparation of subcellular fractions followed by RT-PCR analysis showed better performance of ASOs in nucleus, while both ASOs and siRNAs were able to significantly reduce the cytoplasmic *Tsx* levels (Fig. 3B). Co-localization of *Tsx* and Vimentin confirmed the presence of considerable amounts of *Tsx* signals in the nucleus of siRNAs instead of ASOs (Fig. 3C, indicated by arrows). For the purpose of achieving sufficient generation of phenotype, we imposed a possibly greatest impact of loss of *Tsx* on germ cell differentiation by prolonging post-injection treatment of testis up to 10 days. As a result, *Tsx* levels were suppressed around 60% by ASOs, in contrast with no significance by siRNAs (Fig. 3D). Parallel examination by FISH gave rise to consistent results (Fig. 3E), suggested that ASOs may have more sustainable knockdown effect in vivo than siRNAs.

### *Tsx* inhibition by ASOs raises germ cell defects

**Fig. 4:**
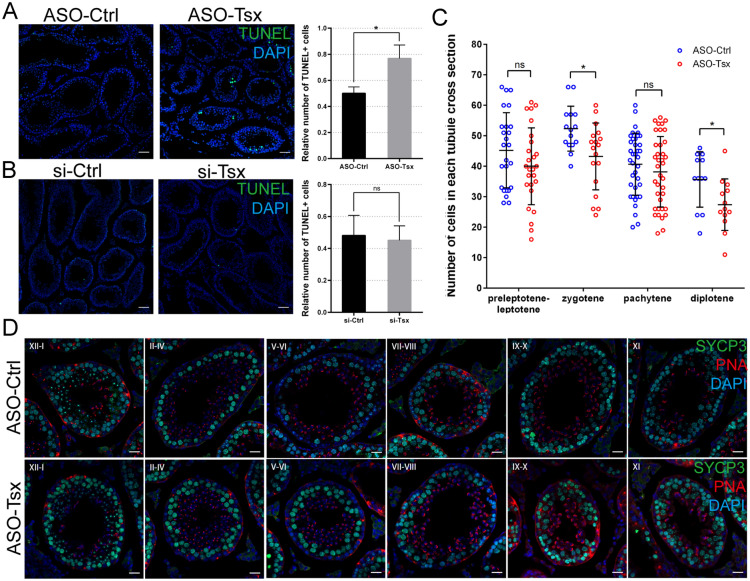
Physiological outcomes of *Tsx* ASOs in testis. **A**, **B** TUNEL staining and quantification of TUNEL+ cells in tubules from *Tsx*-knockdown mice at age of 3 weeks injected with ASOs (**A**) or siRNAs (**B**). Nuclei were stained with DAPI. Scale bar, 50 μm. Relative number of TUNEL+ cells is determined by counting the number of TUNEL+ cells per field and dividing by the number of tubules in each field. 37 fields including 150 tubules from ASOs-mediated *Tsx*-knockdown testes, 31 fields including 125 tubules from ASO-control testes, 21 fields including 100 tubules from siRNAs-mediated *Tsx-*knockdown testes and 30 fields including 111 tubules from siRNA-control were counted. Each contains three independent samples. *p < 0.05. **C** Quantification of four cell populations between control and *Tsx*-knockdown testes. Number of each cell population per tubule is shown in dot. 81 tubules from 3 testes injected with ASO-*Tsx* and 83 tubules from 3 testes injected with control ASO were analyzed. *p < 0.05. **D** Immunostaining of SYCP3 (green) and PNA (red) on testis sections from control and *Tsx*-knockdown mice. Seminiferous tubule sections were staged by morphology of chromosome and acrosome. Scale bar, 20 μm. **A–C** Values are expressed as mean ± SD. Statistical significance was determined using *t* tests

To obtain the stable outcome of *Tsx* loss-of-function during spermatogenesis, we uniformed a workable time window from executing ASOs injection at postnatal day-21, when meiotic spermatocytes are relatively enriched, to ceasing ASO treatment after 10 days, which represents developmental length of the prophase I of meiosis [44]. TUNEL assays showed a statistically significant rise of apoptotic cells in ASO- (Fig. 4A), but not in siRNAs-injected testis (Fig. 4B). Our ASO results coincide with the previous description of *Tsx*-knockout mice [43], supporting ASOs a better tool for *Tsx* knockdown than siRNAs. To investigate whether *Tsx* plays a role in meiotic progression, we counted the average numbers of four types of spermatocytes during meiotic prophase I in each seminiferous tubule. Each cell type can be identified based on its correspondence to the specific stages of seminiferous cycle by co-staining of SYCP3, a marker for core composition of synaptonemal complex, and PNA, a marker for acrosome (Fig. 4C, D). Compared with control, ASO injection resulted in a statistically significant reduction of zygotene and diplotene spermatocytes (Fig. 4C), albeit no stage exhibited evident morphological abnormality (Fig. 4D). Further, we asked whether loss of *Tsx* affected meiotic chromosome synapsis and recombination. Various stages of spermatocytes were identified through chromosome spread analysis via co-staining of SYCP1 and SYCP3. No significant defects were observed in the synapsis of homologous chromosomes (Additional file 1: Fig. S4A). Staining of MLH1 which marks the site of crossover at late pachytene also showed no difference (Additional file 1: Fig. S4B, C), neither was the change indicated by the number of SOX9-positive Sertoli cells where most *Tsx* molecules reside (Additional file 1: Fig. S5A, B). Thus, *Tsx* seems not required for the normal progression of spermatogenesis, whereas it plays a certain role in the balanced maintenance of germ cells among different types as well as their viability.

 To further examine the functional consequence of spermatogenesis from mild defects of germ cells, we evaluated the potential effects on litter size resulting from ASOs-mediated *Tsx* knockdown. Prior to mating test, we prepared another cohort of mice microinjected either with ASO-control or ASO-*Tsx* at 6-week-old. After 10 days of the post-surgery recovery, we performed mating test by either crossing control males or *Tsx*-knockdown males with adult wild-type females. Male fertility can be tested when mice reach 7-8 weeks old when germ cells differentiate into mature sperm, explaining the fertility of these animals. Moreover, we added two stringent control groups (surgical group and non-surgical group) to exclude the potential side-effects of surgical procedures on the litter size, although this problem might be minimized through the post-surgery recovery. RT-PCR results demonstrated that the transcript levels of lncRNA *Tsx* was significantly reduced in males microinjected with ASO-*Tsx* at 6-week-old, compared to that of control group (Additional file 1: Fig. S6A). Further analysis of lncRNA *Tsx* by FISH confirmed a decrease in the expression of lncRNA *Tsx* (Additional file 1: Fig. S6B). Moreover, TUNEL assay revealed an increase in germ cells undergoing apoptosis (Additional file 1: Fig. S6C, D). As expected from the previous genetic lncRNA *Tsx* knockouts [43], males with *Tsx* knockdown through ASO microinjection were able to father litters and displayed normal mating behavior with no effect on the litter size or sex ratios (Additional file 1: Fig. S6E, F). These experiments demonstrated that mice with ASOs-mediated *Tsx* knockdown exhibits defects in germ cell, which is phenotypically similar to the lncRNA *Tsx* knockouts, although either ASOs-mediated *Tsx* knockdown or *Tsx* knockout has no apparent effects on litter size. It’s because the phenotype observed in *Tsx* knockout is mild, evidenced by the abundant sperm present in *Tsx*-deficient mice. It is understandable that the remaining sperm in mice either with *Tsx* knockdown or *Tsx* knockout is sufficient to produce litters. Nonetheless, our ASOs-based in vivo knockdown approach technically enables a knockout-comparable, complete function study of lncRNA in mouse testis.

## Discussion

It remains mysterious how many lncRNAs act usefully in spermatogenesis. In *Drosophila*, large-scale knockout screening has yielded dozens of lncRNAs with visual spermatogenic defects [45]. In mice, however, such strategy is neither practicable nor worthy, setting aside that not all types of lncRNAs can be specifically or completely knocked out [22, 46]. Few reports have identified functional lncRNAs via creation of individual knockout mouse models [43] or using shRNA-mediated knockdown [14]. Unlike shRNA, ASO injection avoids intricate procedures of virus packaging. Common administration ways for ASOs such as intravenous fusion and subcutaneous injection allow for repeated administration [35], which is impractical for seminiferous injection in testis. While our understanding of the in vivo lncRNA physiology stays at its infancy, here we have developed a protocol for microinjecting testis with ASOs to interrogate the function of any individual lncRNAs of interest.

Taking *Tsx* as an example, ASOs were demonstrated to execute high knockdown efficiency in 3-week-old testis so that testicular cells were largely depleted of its signals and exhibited an induction of apoptosis resembling the described *Tsx* knockout [43]. Analysis of *Tsx* levels in subcellular fractions showed that ASOs have better performance than siRNAs in knocking down the nucleus-localized lncRNAs, due possibly to the higher accessibility of ASOs to the nucleus [36], and its unique capability of targeting lncRNAs being transcribed [37]. Compared to siRNAs, moreover, ASOs sustained its effect till post-injection 10 days, a duration long enough to ensure adequate phenotypic analysis. These comparative evaluations support ASOs a better choice for making a knockdown model of lncRNA in living testis. In our study, this *Tsx*-deficiency testis model was used to view the alteration of *Tsx* levels in specific cell types and in different subcellular compartments by FISH and IF double staining. *Tsx* was previously reported to be distributed at the periphery of the tubule where Sertoli cells usually locate by In situ hybridization (ISH) [42]. We found that *Tsx* is a nucleocytoplasmic lncRNA mainly expressed in Sertoli cells and minorly in germ cells. This model was also used to investigate the meiotic defect by employing standard immunofluorescence analyses of either seminiferous sections or chromosome spreads. Our data revealed the involvement of *Tsx* in maintaining a balanced pool of spermatocytes. Although mating test results did not reflect the impact of *Tsx* on the differentiating germ cells, the establishment of knockdown effectiveness in 6-week-old testis as well as exclusion of possible side-effect on mating behavior posed by surgical operation itself has broadened the range of functional assessment of lncRNA to cover male fertility assay. In sum, ASOs-based lncRNA-targeting method, in junction with various follow-up experiments, will facilitate a cascade of investigations to understand the lncRNAs’ roles in mammalian male reproduction.

In recent years, RNA therapeutics based on siRNAs, ASOs and CRISPRi (CRISPR interference) that target transcripts of the genes have emerged. CRISPRi is developed from CRISPR system that has been successfully used in preclinical animal tests and ex vivo modification of iPSCs for transplantation [47–49]. Compared to the infancy of clinical application of CRISPRi, drugs based on siRNAs and ASOs have emerged and been reported to be used in phase III trials [50–52]. Specifically, unlike siRNAs which need a delivery agent, ASOs can be taken up by cells without any delivery agents and has lower biological toxicity. These merits make ASOs more applicable in clinical trials [53–55]. Besides, ASOs can act in the nucleus and intervene in transcription, which makes ASOs widely used in pre-clinical studies of single-gene disorders [56]. The method we established for ASOs-mediated testis knockdown in vivo also offers possibility for pre-clinical application of ASOs in humans with regard to male contraception or improvement of reproductive health [57–59].

## Conclusions

Here we have established an ASO microinjection-initiated procedure that is capable of creating a testis-specific knockdown model for lncRNA. Using this model, along with other experiments, we show that: (1) ASOs efficiently knocked down both the lncRNAs *Malat1* and *Tsx*; (2) *Tsx* is a nucleocytoplasmic lncRNA in testicular cells; (3) *Tsx* can be efficiently and persistently acted upon by ASOs rather than siRNAs; (4) *Tsx* deficiency led to a disturbance to the maintaining of meiotic germ cell pool.

## Materials and methods

### Mice, ASOs, siRNAs and antibodies

C57BL/6 mice were obtained from the Model Animal Research Center of Nanjing University. All mice were housed with 12/12 h light/dark cycles, at 22 °C and allowed free access to water and food.

ASOs and siRNAs were synthesized by Ribobio (Guangzhou, China). Specific sequences are provided in Additional file 2: Table S1. ASOs used in Fig. 1C were diluted with nuclease-free water to different concentrations within 20-500 µM, and injected into the testes of 3-week-old mice with the maximum injection volume (4 µL, with total amount of 1 nmol ASO). Injection volume was increased to 6 µL with total amount of 2.5 nmol ASO when we operated on 6-week-old mice. siRNAs we used in this article were chemically modified with 2’-OM (2-methoxyethyl) and cholesterol to improve the stability and biodistribution [53].

Primary antibodies used in this article are listed as follows: rabbit anti-Vimentin (ab92547, Abcam, USA), rabbit anti-SOX9 (AB5535, Merck Millipore, German), rabbit anti-DDX4 (ab13840, Abcam, USA) and rabbit anti-SYCP3 (ab15093, Abcam, USA), rabbit anti-SYCP1 (ab15090, Abcam, USA), rabbit anti-MLH1 (550,838, BD, USA).

### In vivo knockdown through microinjection

Microinjection of ASOs and siRNAs were operated as previously described [60]. Mice of 3-week-old were anesthetized by tri-bromoethanol and one testis was exteriorized through incisions on abdomen. A mixture of 3 µL (5 µL for 6-week-old mice) of ASO dilutions or siRNA dilutions and 1 µL tracer Trypan blue was injected into seminiferous tubule through the microinjection apparatus (FemtoJet 4i, Eppendorf) under a stereoscopic microscope. The testis was placed back to the abdominal cavity after injection. After both sides of testes were finished with injection, the incisions were closed with sutures. Each injected mouse was kept warm by putting hot water bag in cage until they wake up.

### RNA extraction and RT-PCR

Testes were collected 48 h or 10 days after injection to measure knockdown efficiency. Total RNA was extracted using TRIzol reagent (Thermo Fisher Scientific, USA) following the manufacturer’s instructions. 1 µg RNA was reversed transcribed into cDNA with PrimeScript RT Master Mix (RR036A, TaKaRa, Tokyo, Japan) according to the manufacturer’s instructions. Real time PCR was performed using TB Green *Premix Ex Taq* II (RR820A, TaKaRa, Tokyo, Japan) on Applied Biosystems StepOnePlus Real-Time PCR System. Relative expression of target RNA was determined after normalization to 36B4 gene. The sequences of all primers used in this experiment are provided in Additional file 2: Table S1.

### Rapid amplification of cDNA ends (RACE)

Total RNA was extracted from C57BL/6 mouse testis. 5′ RACE and 3′ RACE were performed using the SMARTer® RACE 5′/3′ Kit (Clontech, Mountain View, CA) according to the manufacturer’s instructions. The following gene-specific primers (GSP) are used for PCR:

5′-TGGCAAGCAACAAACACCCTAGTTGGC-3′ (5′ RACE GSP);

5′-CTTGGGTATCAGCTCCACCAACAAGGT-3′ (3′ RACE GSP).

### Fluorescence in situ hybridization (FISH) and immunofluorescence

Testes were fixed in 4% PFA overnight at 4 °C, dehydrated by graded ethanol (70%, 95%, 100%) and embedded in paraffin. Embedded testes were sectioned into 5 μm. FISH was performed with RNAscope^@^ multiplex fluorescent reagent kit (Advanced Cell Diagnostics, USA) as manufacturer’s instructions [61]. Target probes were obtained from RNAscope^@^. After the last procedure of FISH, sections were treated in 1×PBS for 5 min and then blocked in blocker (10% FBS, 1% BSA, 1% Triton X-100, 0.05% Tween-20) for 1 h at room temperature (RT). Testis sections were incubated with the following antibodies at 4 °C overnight: rabbit anti-Vimentin (1:250), rabbit anti-SOX9 (1:400), rabbit anti-DDX4 (1:200) and rabbit anti-SYCP3 (1:100). Texas red or FITC-conjugated secondary antibodies (Vector Laboratories, USA) were incubated at 37 °C for 1 h and sections were washed 3 times in PBST (1×PBS containing 0.2% Tween-20). Samples were mounted in microslide shield with DAPI. Fluorescent signals were detected on a confocal microscope (LSM800, Carl Zeiss, German).

### Isolation of nuclear and cytoplasmic fractions

Subcellular extracts were prepared as described [60]. 30-40 mg testis tissue was homogenized in 400 µL Cytoplasmic Extraction Buffer [CT Buffer, 250 mM sucrose, 10 mM Tris-HCl (pH 8.0), 10 mM MgCl_2_, 1 mM EGTA, 1× protease inhibitor cocktail III, 0.4 µL RNasin (N251B, Promega, USA)] with 100 strokes. The lysis was centrifuged at 300×*g* for 5 min, and the supernatant was collected as cytoplasmic fraction. Add 1 mL TRIzol reagent per 200 µL supernatant and stored at − 80 ℃. The pellet seen as nuclei fraction was washed three times in 500 µL CT Buffer and centrifuged at 300×*g* for 5 min. Add 1 mL TRIzol and homogenize the pellet by 1 mL syringe with 0.4 mm needle. RNA was extracted as above.

### TUNEL assays, chromosome spread and immunofluorescence

TUNEL was performed on the testis sections with TUNEL BrightGreen Apoptosis Detection Kit (A112-01, Vazyme, China) according to the manufacturer’s instructions.

Chromosome spread of prophase I spermatocytes were performed as previous described [62]. The following primary antibodies were used in morphology analysis of chromosome: rabbit anti-SYCP1 (1:100), rabbit anti-SYCP3 (1:100), rabbit anti-MLH1 (1:100). Slides were washed in 1×PBS for 5 min, and then blocked with 10% goat serum in PBST (1×PBS containing 0.1% Tween 20 ) for 1 h at RT. After incubated with primary antibodies overnight at 4 °C, slides were treated with PBST for 3 times. Secondary antibodies were incubated at 37 °C for 1 h. Wash slides in PBST for 3 times. All the slides were mounted in microslide shield with DAPI. Immunofluorescence for all samples was examined under laser scanning confocal microscope (LSM800, Carl Zeiss, German).

### Mating test

Twenty-one mating cages were divided into three mating groups: Wild-type (WT) males with WT females, ASO-control males with WT females, and ASO-*Tsx* males with WT females. Surgical operation was performed on males at 6-week-old. ASOs-injected males were housed with two WT females after 10 days of post-surgery recovery.

### Statistical analysis

All values were presented as mean ± SD. Statistical analysis was performed with Student’s *t* test (*p < 0.05; **p < 0.01; ***p < 0.001) using Prism 7.0 (GraphPad Software, La Jolla, CA, USA). NS means not significant.

## Supplementary Information

**Additional file 1: Fig. S1.** FISH images of Malat1 on GC-2 cells. **Fig. S2.** 5′ and 3′ RACE results. **Fig. S3.** *Tsx* expression in germ cells. **Fig. S4.** Analysis of impact of *Tsx* deficiency on meiosis. **Fig. S5.** Analysis of impact of *Tsx* deficiency on Sertoli cells. **Fig. S6.** Functional impact of *Tsx* knockdown on male fertility.

**Additional file 2: Table S1.** Sequences of oligonucleotides used in this study.

## Data Availability

All data generated or analyzed during this study are included in this published article and its additional information files.
