# Supplementary material for: Microinjection of antisense oligonucleotides into living mouse testis enables lncRNA function study
Source: Cell Biosci. 2021 Dec 17;11:213. doi: 10.1186/s13578-021-00717-y (PMC8684201; doi:10.1186/s13578-021-00717-y)
Supplement: Supplementary file 1 — Additional file 1: Fig. S1. FISH images of Malat1 on GC-2 cells. Fig. S2. 5′ and 3′ RACE results. Fig. S3. Tsx expression in germ cells. Fig. S4. Analysis of impact of Tsx deficiency on meiosis. Fig. S5. Analysis of impact of Tsx deficiency on Sertoli cells. Fig. S6. Functional impact of Tsx knockdown on male fertility. [file 13578_2021_717_MOESM1_ESM.pdf]

Additional file 1:

Supplementary Figures and Legends S1-S6

Microinjection of Antisense Oligonucleotides into Living Mouse Testis Enables LncRNA Function Study

Zhaohui Chen[1,4], Li Ling[1,4], Xiaolian Shi[1,4], Wu Li[1,4], Huicong Zhai[1,4], Zhenlong Kang[1], Bangjin Zheng[1], Jiaqi Zhu[1], Suni Ye[1], Hao Wang[1], Lingxiu Tong[1], Juan Ni[2], Chaoyang Huang[3]*, Yang Li[1]*, Ke Zheng[1]*

[1]State Key Laboratory of Reproductive Medicine, Nanjing Medical University, Nanjing 211166, China;

[2]Department of Obstetrics and Gynecology, the Affiliated Hospital of Hangzhou Normal University, Zhejiang, 310015, China;

[3]Department of Cardiology, the First Affiliated Hospital, Zhejiang University School of Medicine, Zhejiang, 310014, China;

[4]These authors contributed equally to this work;

*Correspondence: kezheng@njmu.edu.cn, li_yang@njmu.edu.cn, wzy7512@zju.edu.cn

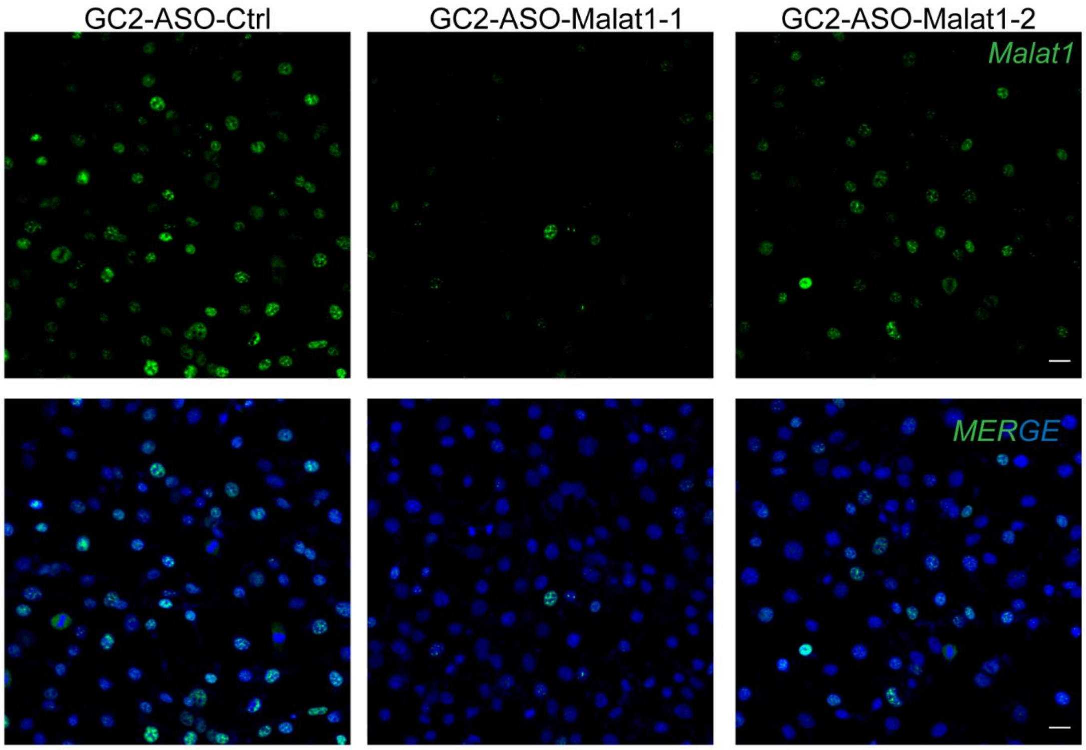

Supplementary Figure 1. FISH images of *Malat1* in GC2 cells transfected with different ASOs. Nuclei was stained in DAPI. Scale bar, 20 μm.

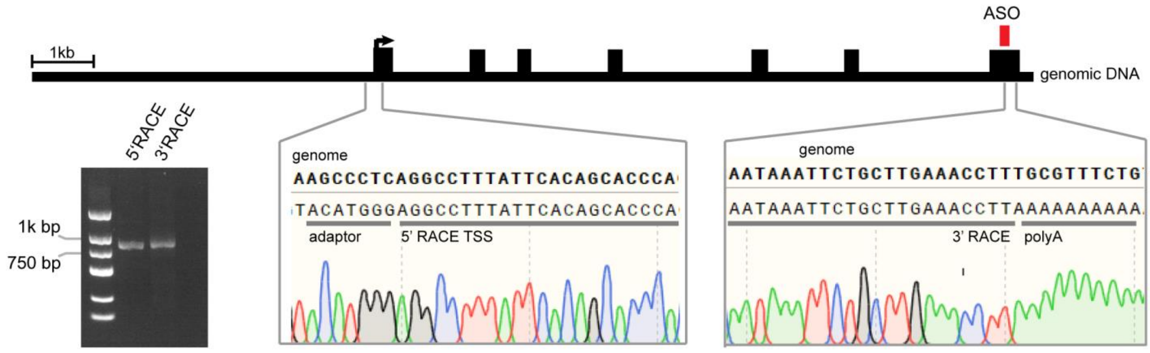

sequence of 5’ RACE

ACATGGGAGGCCTTTATTCACAGCACCCACCTAGACTTGGGTATCAGCTCCACCAACAAG-
GTCTCCCTCCACAGAAGCCGAATCCCGGAGCTATGTCTGAAAAGCAAAGCCCCAAGACCTCTGAA
GCAGAATGCAGTGCAATGGACTTGCCAGAATTCGAGGATGAAGAAAACTGGCTTTTCAAAGTTCT
GGGATTCCAGCCTGGGCCGTCCTCTGCTCTGGATGATGACACCGACGATCAGGCAGATGAGCCA
CTGAGCGCTGCCGAATTTCTTCACCTGCAAGATATTCTTCAGGAGGACAGAGTCAGCAGTACCGA
TGATGAGGACACTTGCCAAGCTGGATGTACTGAAGATGATGAAACCAGTCACAGTGACAGAGACA
TAGACAATAATGTGAAAGTCATCACTGGCAACATTAAAGCAAGCCCCTCCATGTATATGGAGATGTT
CACTGATCAGAACCCACAAGCTGACCAAGACCTGGAAGAAACTGAATCAGATGGTGCCATGAACC
CAACTGATTAAGCAGGCAGGCAGAAACGTGTCACCATCAGCTACCATTGTGCCCTCATGCATAAAT
GAAGTCCTGATGGAAAACAGACAAACAAACAAACAAACAAACATCTCTACACACTAAGCCGAAAAC
AAATCTATACCAAACTGCTAAAATAAATGGAAAATACTTAATTTGAGCCTGGCTGTTTTGAAAATTGG
AAATCCTTCTTCAAAATGAAAATCACCGCAAAACAATCTAAAGTCATCTACAGGCCAACTAGGGTGT
TTGTTGCTTGCCA

sequence of 3’ RACE

CTTGGGTATCAGCTCCACCAACAAGGTCTCCCTCCACAGAAGCCGAATCCCGGAGCTAT-
GTCTGAAAAGCAAAGCCCCAAGACCTCTGAAGCAGAATGCAGTGCAATGGACTTGCCAGAATTCG
AGGATGAAGAAAACTGGCTTTTCAAAGTTCTGGGATTCCAGCCTGGGCCGTCCTCTGCTCTGGAT
GATGACACCGACGATCAGGCAGATGAGCCACTGAGCGCTGCCGAATTTCTTCACCTGCAAGATAT
TCTTCAGGAGGACAGAGTCAGCAGTACCGATGATGAGGACACTTGCCAAGCTGGATGTACTGAAG
ATGATGAAACCAGTCACAGTGACAGAGACATAGACAATAATGTGAAAGTCATCACTGGCAACATTAA
AGCAAGCCCCTCCATGTATATGGAGATGTTCACTGATCAGAACCCACAAGCTGACCAAGACCTGGA
AGAAACTGAATCAGATGGTGCCATGAACCCAACTGATTAAGCAGGCAGGCAGAAACGTGTCACCA
TCAGCTACCATTGTGCCCTCATGCATAAATGAAGTCCTGATGGAAAACAGACAAACAAACAAACAA
ACAAACATCTCTACACACTAAGCCGAAAACAAATCTATACCAAACTGCTAAAATAAATGGAAAATACT
TAATTTGAGCCTGGCTGTTTTGAAAATTGGAAATCCTTCTTCAAAATGAAAATCACCGCAAAACAAT
CTAAAGTCATCTACAGGCCAACTAGGGTGTTTGTTGCTTGCCAAACTTCCAATAAATTCTGCTTGAA
ACCTTAAAAAAAAAAAAAA

Supplementary Figure 2. 5’ and 3’ RACE results. Relative localization of exons of *Tsx* on genome is marked by black boxes on the black line. The red vertical line refers to the relative localization of ASO-*Tsx*-2 sequence. Below the diagram, left: gel electrophoresis image of PCR products from the 5′ RACE and 3′ RACE assays; right: Sanger results of 5′ RACE and 3′ RACE mapped to the genome of *Tsx*. The detailed sequences of 5’ RACE and 3’ RACE are shown below.

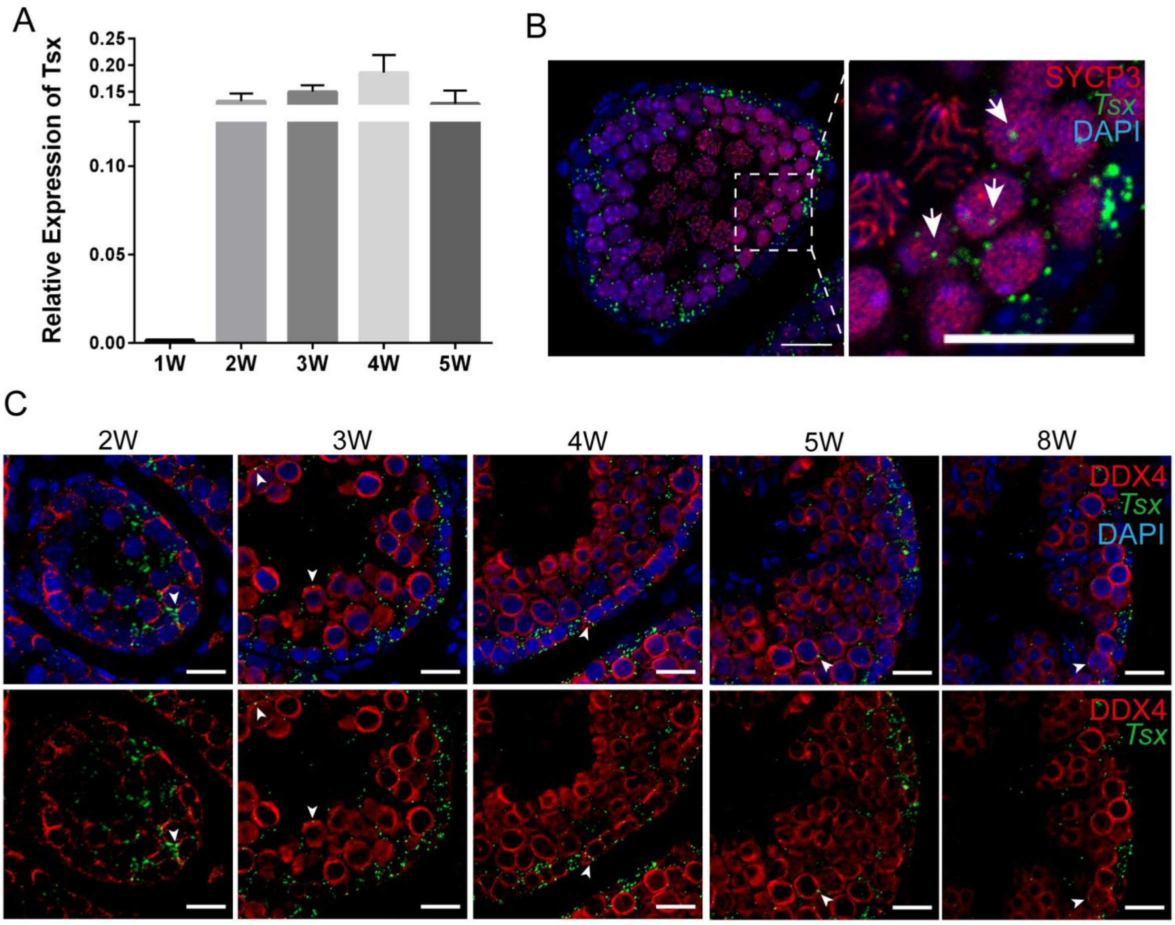

Supplementary Figure 3. *Tsx* expression in germ cells. (A) RT-PCR analysis of *Tsx* RNA expressed in mice testes at different ages. n=3. Values were expressed as mean±SD. (B) FISH of *Tsx* RNA (green) with SYCP3 (red) of 2-week-old mouse testis section. *Tsx* signals located in nuclei of SYCP3-positive cells are indicated by white arrows. Scale bar, 20 μm. (C) FISH of *Tsx* RNA (green) with DDX4 (red) staining on testis sections from different week-old mice. Nuclei were stained with DAPI. *Tsx* signals overlapped with DDX4 staining area are indicated by white arrows. Scale bar, 20 μm.

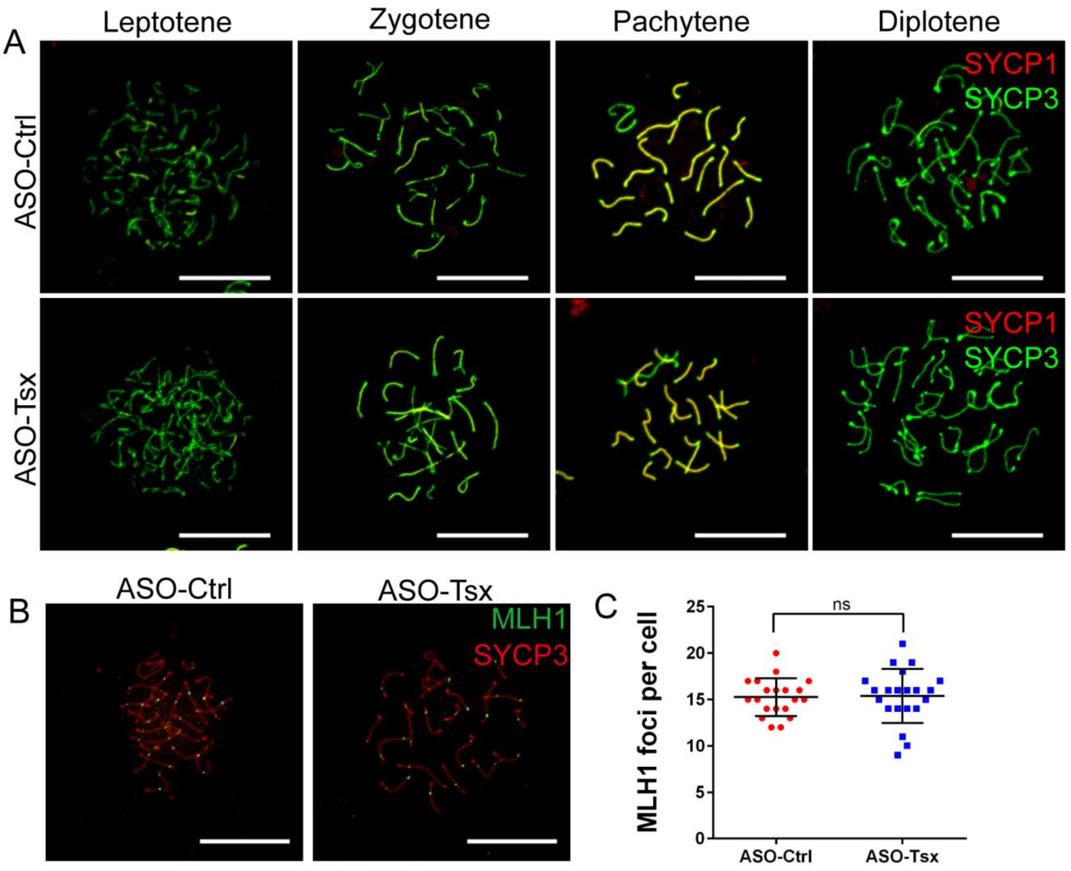

Supplementary Figure 4. Analysis of impact of *Tsx* deficiency on meiosis. (A) Double Immunostaining of SYCP1(red) and SYCP3(green) on chromosome spread samples from *Tsx*-knockdown testis collected 10 days after injection. Scale bar, 20 μm. (B) Immunostaining with MLH1(red) and SYCP3(green) of spermatocytes from *Tsx*-knockdown testis collected 10 days after injection. Scale bar, 20 μm. (C) Quantification of MLH1 foci number per pachytene spermatocyte. Each number is shown in dot. Statistical significance was determined using *t* tests.

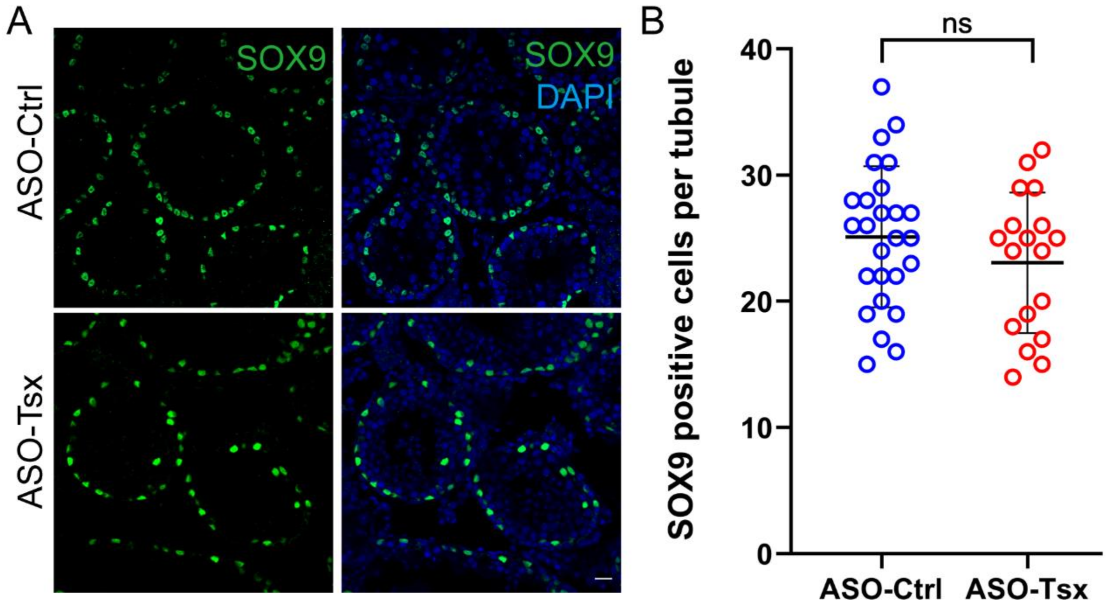

Supplementary Figure 5. Analysis of impact of *Tsx* deficiency on Sertoli cells. (A) Immunostaining of Sertoli cells marker SOX9 (green) on testis sections from *Tsx*-knockdown mouse and control mouse. Scale bar, 20 μm. (B) Quantification of SOX9-positive cells and number of cells per tubule is shown in dot. 18 tubules injected with ASO-*Tsx*-2 and 26 tubules injected with control ASO were analyzed. Values are expressed as mean±S.D. Statistical significance was determined using *t* tests.

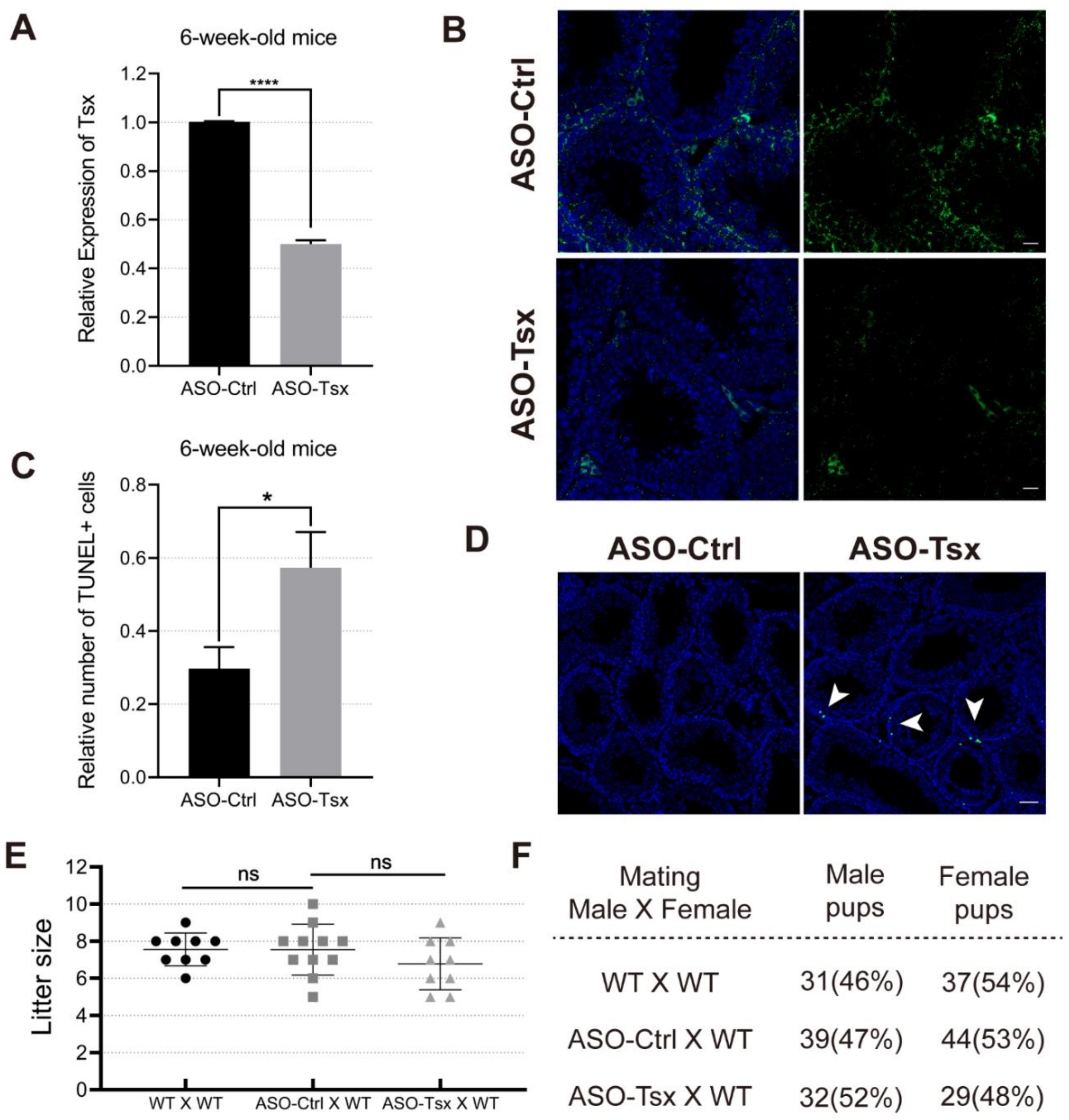

Supplementary Figure 6. Functional impact of *Tsx* knockdown on male fertility. (A) RT-PCR analysis of *Tsx* RNA expressed in control and knockdown testis from 6-week-old mice. ****p<0.0001, n=3. (B) FISH images of control and *Tsx*-knockdown testes from 6-week-old mice. Scale bar, 20 μm. (C) Quantification of TUNEL+ cells in tubules from 6-week-old *Tsx*-knockdown testis. A total of 29 fields including 140 tubules from *Tsx*-knockdown testes and 21 fields including 124 tubules from control testes were counted. Each contains three independent samples. Values are expressed as mean±S.D. Statistical significance was determined using *t* tests. *p<0.05. (D) TUNEL staining of apoptotic cells in tubules from *Tsx*-knockdown mice injected with ASOs. TUNEL+ cells were indicated by white arrows. Nuclei were stained with DAPI. Scale bar, 50 μm. (E) Mating test of *Tsx*-knockdown mice. Numbers of pups per litter were shown as dots. Values are expressed as mean±S.D. Statistical significance was determined using *t* tests. (F) Sex ratios of offspring in mating test.
