# Supplementary material for: Microinjection of antisense oligonucleotides into living mouse testis enables lncRNA function study
Source: Cell Biosci. 2021 Dec 17;11:213. doi: 10.1186/s13578-021-00717-y (PMC8684201; doi:10.1186/s13578-021-00717-y)
Supplement: Supplementary file 2 — Additional file 2: Table S1. Sequences of oligonucleotides used in this study. [file 13578_2021_717_MOESM2_ESM.pdf]

Table S1. Sequences of oligonucleotides used in this study

**Name**	**Sequence (5’ to 3’)**
Tsx forward primer	TGACCAAGACCTGGAAGAAACT
Tsx reverse primer	TCTGTTTTCCATCAGGACTTCA
Malat1 forward primer	GTTACCAGCCCAAACCTCAA
Malat1 reverse primer	CTACATTCCCACCCAGCACT
36B4 forward primer	GCAGATCGGGTACCCAACTGTTG
36B4 reverse primer	CAGCAGCCGCAAATGCAGATG
ASO-*Malat1*-1	GAAGTTTGCATGTACGCGGT
ASO-*Malat1*-2	ATGACCGACGTAGTTCCACT
ASO-*Tsx*-1	AGTAGACGGACTAGCAGCCA
ASO-*Tsx*-2	AAACAAAAGCCGAATCACAC
ASO-*Tsx*-3	TAACAGATACAGAGACAGTG
si-*Tsx*-1	UGAUUCAGUUUCUUCCAGG
si-*Tsx*-2	UUGUUUUCGGCUUAGUGUG
si-*Tsx*-3	AUUGUCUAUGUCUCUGUCA
